# The public’s belief in climate change and its human cause are increasing over time

**DOI:** 10.1371/journal.pone.0174246

**Published:** 2017-03-20

**Authors:** Taciano L. Milfont, Marc S. Wilson, Chris G. Sibley

**Affiliations:** 1 School of Psychology, Victoria University of Wellington, Wellington, New Zealand; 2 School of Psychology, University of Auckland, Auckland, New Zealand; Universitat Trier, GERMANY

## Abstract

Polls examining public opinion on the subject of climate change are now commonplace, and one-off public opinion polls provide a snapshot of citizen's opinions that can inform policy and communication strategies. However, cross-sectional polls do not track opinions over time, thus making it impossible to ascertain whether key climate change beliefs held by the same group of individuals are changing or not. Here we examine the extent to which individual's level of agreement with two key beliefs ("climate change is real" and "climate change is caused by humans") remain stable or increase/decrease over a six-year period in New Zealand using latent growth curve modelling (n = 10,436). Data were drawn from the New Zealand Attitudes and Values Study, a probabilistic national panel study, and indicated that levels of agreement to both beliefs have steadily increased over the 2009–2015 period. Given that climate change beliefs and concerns are key predictors of climate change action, our findings suggest that a combination of targeted endeavors, as well as serendipitous events, may successfully convey the emergency of the issue.

## Introduction

In 2015, Pope Francis encouraged Catholics worldwide to be attentive to global climate change in his encyclical *Laudato si’*. More recently, Leonardo DiCaprio was allowed a longer-than-average Oscar acceptance speech to similarly raise worldwide attention to climate change, while leaders and representatives from over 150 countries gathered to sign the global climate change agreement reached in Paris in December, 2015. One question that arises from these international examples of public figures trying to promote awareness and action on climate change is whether they reflect and/or influence a popular upswing in climate change beliefs and concerns over time.

Although perceptions of climate change threat and beliefs vary internationally [1], opinion polls indicate that an overall global majority believe in the reality and human causation of climate change [2–4]. In particular, short-term longitudinal panel studies that have examined the effect of factors such as popular media interventions or extreme weather events on climate change concern suggest that individuals’ attitudes may change after exposure to such events. For example, a field experiment investigating impacts of the provision of climate information through TV meteorologists (presented on air and online) indicated that viewers were both more concerned and more likely to perceive scientific consensus than others [5]. Another study found that climate change concern was greater among UK respondents who have had first-hand experience with floods ([6]; see also [7]). In contrast, little change in climate change attitudes were observed pre- and post-2012 Midwestern US drought [8], and UK viewers of the climate change documentary *The Age of Stupid* initially reported increased concern, action intention and efficacy but these effects deteriorated over time [9].

Although inconclusive, this emerging literature suggests that researchers and policy makers would benefit by knowing whether citizens are becoming more or less credulous of climate change today than previously, and also the rate of change in citizens’ belief in climate change. Public opinion polling on the subject of climate change concern and beliefs is now common-place, and results from such one-off surveys are useful in providing a snapshot of the level of agreement and/or support to climate-related questions and issues from a given population. However, such cohort-based findings are limited in the extent to which researchers and practitioners can draw inferences about the nature, and causes, of changes in attitude or belief. To illustrate, while results suggest that climate change concern among Americans increased significantly during the 1980s, more recent polls suggest that fewer American citizens consider climate change to be a threat than only a few years prior [2]. Since these studies base their conclusions on distinct population cohorts, we do not have a particularly robust understanding of whether, and how much, beliefs about global climate change are indeed changing over time. Thanks to the ubiquity of public opinion polls, we also know that perception of climate change threat varies internationally [1] and fluctuates over time [2, 10].

Studies assessing climate-relevant questions longitudinally are better equipped than cross-sectional public opinion polls to provide information regarding temporal change in climate change attitudes and beliefs. However, there are relatively few studies assessing climate-relevant attitudes in the same panel longitudinally, and very few indeed that do so over more than a year, or with a representative sample.

One study assessed a national sample of 269 New Zealanders’ responses on climate-related questions three times over a one-year period [11], reporting that concern about global warming and climate change did not increase over this duration—perhaps due to the limited time frame—but that higher levels of climate-related knowledge increased climate change concern, which in turn translated into greater subjective environmental responsibility. Another study assessed a nationally representative sample of US citizens about climate belief and personal experience of climate and weather, assessed in 2008 and 2011 [12]. Results showed that personal experience of climate change-affected weather increased climate change belief and certainty, but also that high certainty also predicted subjective experience—strong climate change skeptics and believers tended to interpret subjective weather experience consistent with their belief.

Another large scale study with a longitudinal component reported that a majority of Australians believe climate change is occurring, that climate change is of lesser priority than other personal concerns, and that Australians were poor predictors of others’ attitudes; but longitudinal analyses are not reported excepting in reference to absolute levels of attitude change over time [13]. Another longitudinal study conducted in Australian provides a qualitative analysis of perceptions of climate and climate change, emphasising that for many people the notion of “natural cycles” was an important common sense idea that served to provide an appreciation of an understandable dynamic relationship between humans and their environment [14].

Despite the increase in the number of studies examining change in climate change-related variables across time considering the same population, none of the available studies are both representative or of a sufficiently large scale to draw and report robust inferences of population change. In response to these challenges, we present the first longitudinal analysis of key climate change beliefs with a large nationally representative sample assessed over a six-year period (from 2009 to 2015). The aim of this research is to present an initial flavour of the extent and rate of change of climate change beliefs. We analyse the first six waves of longitudinal data from the New Zealand Attitudes and Values Study comprising 10,436 respondents who completed at least three of the waves of annual data collection. Participants indicated their level of agreement to one question examining the belief in the reality of climate change and another question examining belief in anthropogenic climate change.

## Methods

### Sampling procedure

This study analysed data from the New Zealand Attitudes and Values Study (NZAVS), which is is an annual, longitudinal national probability study that has been assessing people’s socio-political attitudes annually since 2009. Invitations to participate in a mail-based survey were sent to people randomly sampled from the New Zealand Electoral Roll. Participants were posted a copy of the questionnaire, with a second postal follow-up two months later. Participants who provided an email address in a previous wave were also emailed and invited to complete an online version if they preferred.

The NZAVS is reviewed every three years by the University of Auckland Human Participants Ethics Committee. The first phases of the longitudinal study were approved on 09-September-2009 for 3 years (reference number: 2009/336). Ethics approval for the study was re-approved by the University of Auckland Human Participants Ethics Committee on 17-February-2012 until 09-September-2015 (reference number: 6171), and then on 03-June-2015 until 03-June-2018 (reference number: 014889). All participants granted informed written consent. Contact details are removed when the questionnaires are received, and all data were de-identified before analyses were conducted. NZAVS data is hosted at the University of Auckland, and the de-identified data is available to appropriately qualified researchers upon request for the purposes of re-analysis. We describe in more detail the sampling procedure for each of the waves used in this study in S1 Supporting Information (see also [15]).

### Participants

Table 1 presents sample size and characteristics for each of the survey waves (see also S1 Supporting Information). The present analysis included only participants who responded to three or more waves. Missing data among those completing at least three waves was estimated using Full Information Maximum Likelihood and assuming that data were missing at random. This assumption seems reasonable given that analysis of panel attrition indicates that demographic factors are only weakly predictive of dropout in the NZAVS [15]; however, people for whom English is not a first language are more likely to drop out of the study relative to other groups. This method of missing data estimation weighted each individual-level trajectory based on its reliability, which is in turn a function that takes the number of observations into account. Those who responded to all six-time points contributed the most information to the estimation of the mean-level trajectory, while those who responded to three contributed the least. Syntax for our Mplus models are available on the NZAVS website: www.psych.auckland.ac.nz/uoa/NZAVS

**Table 1:** Distribution of responses to the questions “Climate change is real” (reality) and “Climate change is caused by humans” (cause) in the first six waves of the New Zealand Attitudes and Values Study, with descriptive statistics for each question at each wave.

		Response options									
Year	Question	1 strongly disagree	2	3	4	5	6	7 strongly agree	*M*	*SD*	*N*
2009	Reality	4%	4%	6%	14%	15%	24%	33%	5.34	1.69	4630
	Cause	6	7	9	19	19	22	17	4.74	1.74	4591
2010	Reality	4	5	6	15	17	26	28	5.26	1.65	4022
	Cause	7	7	9	20	20	21	17	4.70	1.74	3993
2011	Reality	3	4	5	15	18	24	31	5.37	1.62	6177
	Cause	6	7	8	19	20	22	17	4.75	1.74	6127
2012	Reality	3	3	5	13	16	26	34	5.45	1.61	8638
	Cause	5	6	9	18	21	22	19	4.84	1.70	8592
2013	Reality	3	3	4	12	15	24	37	5.56	1.58	9188
	Cause	5	6	7	17	19	24	23	5.02	1.69	9172
2014	Reality	2	2	4	11	17	24	40	5.72	1.44	8928
	Cause	3	4	6	17	20	23	26	5.20	1.61	8889

Note: Distributions are based on valid percent, and sample sizes at each time point refer to those who completed at least three of the first six waves of the New Zealand Attitudes and Values Study.

### Measures

The two NZAVS questions analysed in the present article (i.e., “Climate change is real” and “Climate change is caused by humans”) were embedded in a large battery of Likert-type questions. Across the first six waves of the NZAVS respondents expressed their levels of disagreement-agreement to these questions on a 7-point answer scale with end-labels only: 1 (*strongly disagree*) and 7 (*strongly agree*). Table 1 presents the mean and standard deviation for the agreement levels of these questions, as well as the frequency distribution of the answers.

### Analysis

We simultaneously examined respondents’ level of agreement to both climate change beliefs across time using a parallel process Latent Growth Model. This technique has many advantages when compared to other longitudinal data-analytic techniques [16]. Some of the advantages include the ability to estimate reference levels (or initial amount; *intercept*) and their developmental trajectories to and from those levels (*slope*); the ability to estimate change in latent variables as the technique is a special case of structural equation modeling; the ability to estimate means, variances and covariances of individuals differences in both intercepts and slopes; and the technique does not assume that all individuals in a particular longitudinal project change over time at the same rate.

We estimated the rate of change in levels of disagreement-agreement with the two climate change belief items over the October 2009 –October 2015 period. We modelled the intercepts, and linear and quadratic growth factors for these two aspects of climate belief as parallel processes, and allowed the intercepts and slopes to correlate with one another.

To reflect the variation in response time to each wave between individuals, response time at each wave was then converted into yearly units and modelled as individually time-varying effects, with time = 0 being the 30th June, 2009. This accounted for the fact that different individuals completed the surveys at different times throughout the year, and with different durations between each assessment, rather than fixing all responses in 2009 to 1, all responses in 2010 to 2, and so forth. Our models therefore provide a latent intercept representing estimated mean climate change belief at 30th June, 2009. Residual variances, or disturbances, in the manifest ratings of both Likert items were constrained to equality across waves. This therefore assumes that the amount of variation that is unexplained by the intercept and rate of change in our outcome measure is the same across waves [17].

Our model estimated the fixed and random effect for the intercept, linear and quadratic growth components spanning Waves 1–6 of data collection. The intercept represents the mean score on the outcome (disagreement-agreement levels for the climate item of interest) as of June 2009. Belief in climate change reality and human causation were then estimated every three months from October 2009 to October 2015.

We integrated the parameters for the fixed effects in our models to generate the model-implied rate of change in climate beliefs over the October 2009–October 2015 period. The following regression equation was used to estimate this model-implied rate of change:

$$y_{t}=c+st+s^{2}t^{2}$$

In this regression equation, *y*_*t*_ is the predicted level of climate belief in the given question at time *t*, *c* is the intercept, or model-predicted mean level of belief when *t* = 0 (i.e., 30th June, 2009), *s* is the fixed effect for the linear growth component of the model, and *s*^*2*^ is the fixed effect for the quadratic growth component. Values of *t* are distributed in this model so that 1.0 unit represents a change of one year. We used the parameters reported in Table 2 to derive model-implied levels of disagreement-agreement that “climate change is real” and “climate change is caused by humans” for values of *t* ranging from .25 (October 2009) to 6.25 (October 2015) in quarterly increments.

**Table 2:** Fixed and random effects for parallel process Latent Growth Model predicting change in disagreement to agreement (on a scale from 1 to 7) in response to key climate change questions) for New Zealand residents in the first six waves of the New Zealand Attitudes and Values Study over the October 2009 –October 2015 period.

*Model*	“Climate change is real”	“Climate change is caused by humans”
***Linear***		
*Fixed effects (means)*		
Intercept	5.244 (.019)**	4.612 (.020)**
Linear growth parameter	.072 (.003)**	.090 (.004)**
*Random effects (variances)*		
Intercept	2.246 (.059)**	2.409 (.053)**
Linear growth parameter	.031 (.003)**	.030 (.002)**
***Quadratic***		
*Fixed effects (means)*		
Intercept	5.403 (0.023)**	4.813 (0.024)**
Linear growth parameter	-0.061 (0.012)**	-0.080 (0.013)**
Quadratic growth parameter	0.020 (0.002)**	0.026 (0.002)**
*Random effects (variances)*		
Intercept	2.209 (0.070)**	2.396 (0.062)**
Linear growth parameter	0.262 (0.033)**	0.306 (0.031)**
Quadratic growth parameter	0.005 (0.001)**	0.006 (0.001)**

Note: *N* = 10,436 participants who completed at least three of the first six waves of the New Zealand Attitudes and Values Study. Participants who completed less than three of the six waves were excluded from the model. Missing data among participants who completed three or more waves were estimated using Full Information Maximum Likelihood and assuming data were missing at random. Models estimated using Maximum Likelihood with robust estimation of standard errors. Standard errors reported in parentheses. Disturbances of the manifest indicators were constrained to equality over time. Fit indices: Linear model: loglikelihood = -130590.18, AIC = 261212.36, BIC = 261328.41. loglikelihood = -130055.73, AIC = 260169.46, BIC = 260379.79.

*p < .05.

**p < .01.

## Results

**Fig 1:**
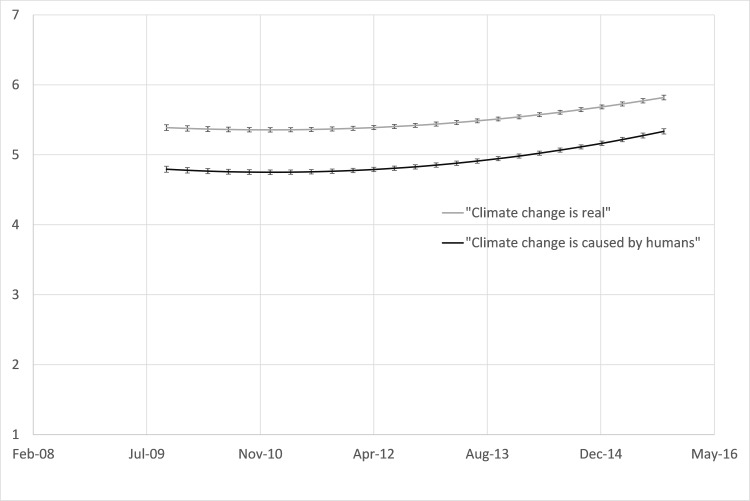
Model-implied rate of change in the level of agreement with climate change beliefs. Rate of change over the 2009–2015 period for 10,436 participants who completed at least three of the first six waves of the New Zealand Attitudes and Values Study. Ratings ranged from 1 (*strongly disagree*) to 7 (*strongly agree*), and error bars represent the 95% CI of the model-implied estimate.

Fig 1 presents the estimated rates of change in disagreement to agreement in response to the climate change belief items over the six-year period. Our results show that agreement with climate change reality was higher at all time points than agreement with anthropogenic climate change.

What about change in the climate change beliefs over time? Table 2 presents the results from the parallel process Latent Growth Model predicting change in disagreement to agreement in response to the climate change belief items. We first tested a linear growth model, and then extended this to include a second-order polynomial (quadratic) growth component. The quadratic growth component in the quadratic model was significant, and thus we focus on this model as it allowed for potential change occurring in a simple accelerating (or decelerating) fashion.

Our analyses reveal a significant curvilinear increase in the average level of agreement with both climate change beliefs over the 2009–2015 period. This curvilinear rate of growth occurred because climate change beliefs were generally increasing over time, but in an accelerating fashion with the most rapid increased occurring from about 2013 onwards (see Fig 1). Moreover, although the observed increase was relatively small, the across-time increase was statistically significant for both belief in climate change reality and anthropogenic climate change. Our results show that from October 2009 to October 2015 respondents’ level of agreement about the reality of climate change increased, on average, from 5.39 to 5.82 on an agreement scale ranging from 1–7. Similarly, respondents’ level of agreement for anthropogenic climate change increased on average from 4.79 to 5.34 for the same six-year period.

Finally, we examined whether the observed increase in average levels of agreement in climate change reality correlated with the observed increase in average levels of agreement in anthropogenic climate change, and vice-versa. In the linear (baseline) model, the linear growth functions for climate change reality and anthropogenic climate change were positively associated (b = .027, se = .002, z = 15.773, p < .001). The linear growth factors for reality and anthropogenic beliefs were also correlated in the quadratic model (b = .232, se = .020, z = 11.51, p < .001), as were the quadratic growth factors (b = .005, se = .001, z = 9.49, p < .001). These results indicate that people who tended to increase their level of agreement in one climate change belief also tended to increase their agreement level in the other belief.

## Discussion

We provide the first comprehensive population-level examination of change in climate change beliefs. First, we showed that the level of agreement to the reality of climate change is greater than agreement to its human causation. This indicates a clear differentiation of the two issues among respondents regarding their belief levels: Respondents in our sample are more likely to believe in the reality of climate change than whether or not climate change is caused by humans. Perhaps believe in the reality of climate change is a precondition for believing in its human causation. This is consequential since the belief in climate change reality has been shown to have stronger effects on a number of outcome variables compared to the belief in anthropogenic climate change; moreover, these beliefs interact so that belief in climate change reality is more predictive for individuals who strongly believe in human cause of climate change [18].

We then showed that agreement to the reality of climate change and agreement to its human causation showed comparable (and coupled) increase over the six-year period. The observed steady increase in climate change belief over a six-year period is promising and suggests that people (at least those in New Zealand) are becoming increasingly aware of the reality of climate change and its human causation. We examine change spanning the 2009–2015 period, and showed that this increase in belief has been most pronounced in more recent years, from about 2013 onwards. It should also be noted that a simple linear model also fit the data fairly well, indicating that the core pattern in our data is a general and gradual increase in climate belief, with the caveat that such change may be slightly more pronounced in recent years. Moreover, that across-time increase in climate change beliefs are related is consequential given that these beliefs predict a number of important outcomes relevant for mitigation and adaptation actions [18, 19].

Past research has examined the many predictors of climate change concern and belief [20–24], with a recent meta-analysis demonstrating that political affiliation and political ideology are the main predictors of climate change belief [19]. Consistent with this finding, previous research in New Zealand identifies a latent profile of respondents (“climate skeptics”) with low agreement levels in both climate change reality and its human causation that had the highest levels of self-reported political conservatism [25]. Since distrust in the reality of climate change and its human cause is associated with a more conservative political orientation, it is likely that change in climate change belief will differ for distinct groups in a given society. That is to say, we predict that the observed increase in climate change beliefs might be greatest among politically liberal individuals, while small or null for their politically conservative counterparts. Due to power considerations we cannot test this prediction at present [26], but the New Zealand Attitudes and Values Study is ongoing and this is something we aim to address in the years to come. Similar to public opinion polls showing that perception of climate change threat fluctuates over time [2] and [10], we also expect that climate change beliefs would fluctuate over time. The ongoing nature of this longitudinal project means we will be able to pinpoint whether fluctuations on climate change beliefs is a direct result of particular socio-economic circumstances.

To conclude, our findings suggest that communicating the reality and urgency of climate change—perhaps combined with media attention to the issue [27] and with communicating individual experience of climate change-related extreme weather events [5]—is successfully influencing the levels of climate change belief in New Zealand. Similar studies will be able to demonstrate whether this is also happening in other societies.

## Supporting information

S1 Supporting Information (DOCX)
