# Supplementary material for: The public’s belief in climate change and its human cause are increasing over time
Source: PLoS One. 2017 Mar 20;12(3):e0174246. doi: 10.1371/journal.pone.0174246 (PMC5358842; doi:10.1371/journal.pone.0174246)
Supplement: S1 Supporting Information — (DOCX) [file pone.0174246.s001.docx]

**Supplementary Material**

As noted in the main article, the New Zealand Attitudes and Values Survey (NZAVS) is a national panel study that has been assessing people’s socio-political attitudes annually since 2009. We describe in more detail the sampling procedure for each of the waves used in this study below.

***Sampling Procedure -- NZAVS Time 1 (2009).*** The Time 1 (2009) NZAVS contained responses from 6,518 participants sampled from the 2009 New Zealand Electoral Roll. The electoral roll is publicly available for scientific research and in 2009 contained 2,986,546 registered voters. This a compulsory list of eligible voters representing all citizens over 18 years of age who were eligible to vote regardless of whether they chose to vote, barring people who had their contact details removed due to specific case-by-case concerns about privacy. The sample frame was spilt into three parts. Sample Frame 1 constituted a random sample of 25,000 people from the electoral roll (4,060 respondents), and Sample Frame 2 constituted a second random sample of a further 10,000 people from the electoral roll (1,609 respondents).

Sample Frame 3 constituted a booster sample of 5,500 people randomly selected from meshblock area units of the country with a high proportion of Māori, Pacific Nations and Asian peoples (671 respondents). Statistics New Zealand (2013) define the meshblock as “the smallest geographic unit for which statistical data is collected and processed by Statistics New Zealand. A meshblock is a defined geographic area, varying in size from part of a city block to large areas of rural land. Each meshblock abuts against another to form a network covering all of New Zealand including coasts and inlets, and extending out to the two hundred mile economic zone. Meshblocks are added together to ‘build up’ larger geographic areas such as area units and urban areas. They are also the principal unit used to draw-up and define electoral district and local authority boundaries.” Meshblocks were selected using ethnic group proportions based on 2006 national census data. A further 178 people responded but did not provide contact details and so could not be matched to a sample frame.

In sum, postal questionnaires were sent to 40,500 registered voters or roughly 1.36% of all registered voters in New Zealand. The overall response rate (adjusting for the address accuracy of the electoral roll and including anonymous responses) was 16.6%.

***Sampling Procedure -- NZAVS Time 2 (2010).***The Time 2 (2010) NZAVS contained responses from 4,442 participants. The Time 2 (2010) NZAVS retained 4,423 from the initial Time 1 (2009) NZAVS sample of 6,518 participants, and included an additional 20 respondents who could not be matched to the Time 1 participant database (a retention rate of 67.9% over one year).

***Sampling Procedure -- NZAVS Time 3 (2011).***The Time 3 (2011) NZAVS contained responses from 6,884 participants, including 3,918 retained from one or more previous wave, 2,962 new additions from booster sampling, and 4 unmatched participants or unsolicited opt-ins. The Time 3 (2011) NZAVS retained 3,915 from the initial Time 1 national probability sample (a 60.1% retention rate over two years). A further three participants who joined at Time 2 were also retained. Again participants were posted a copy of the questionnaire, with a second postal follow-up two months later, or invited to complete an online version if they preferred.

To boost sample size at Time 3 and compensate for sample attrition, a booster sample was recruited through an unrelated survey posted on the website of a major New Zealand newspaper in 2011. A total of 3,208 participants registered an initial expression of interest in being contacted to participate in the NZAVS via this survey. Participants in this non-random booster sample were emailed an invitation to participate in an online version of the NZAVS, and those who did not respond to the email were also sent a postal version of the questionnaire. A total of 2,962 participants completed the questionnaire when subsequently contacted (92.4%).

***Sampling Procedure -- NZAVS Time 3.5 (2012 mid-year).***The Time 3.5 (2012 mid-year) NZAVS contained responses from 4,514 participants who completed a follow-up online-only questionnaire administered roughly six months following the full Time 3 (2011) questionnaire. The Time 3.5 sample was supplementary to the full Time 3 (2011) NZAVS. The sample frame included those participants who had provided an email address when completing the full Time 3 questionnaire earlier that year, as well as approximately 400-450 Pacific participants who were recruited informally via Pacifika networks.

The sample included 1,977 retained participants from the initial Time 1 (2009) NZAVS random electoral roll sample, 2,113 participants from the non-random online newspaper website sample collected as part of the Time 3 (2011) NZAVS, a further 50 participants online newspaper website sample who has initially indicated but had not responded initially at Time 3 (but did responded when contacted again at Time 3.5), 197 participants who self-selected into the study or who were unable to be matched to a sample frame, and 177 additional Pacific participants recruited via Pacifika networks who complete a different version of the questionnaire focusing on Pacific identity and wellbeing.

***Sampling Procedure -- NZAVS Time 4 (2012).*** The Time 4 (2012) NZAVS contained responses from 12,182 participants, including 6,807 retained from one or more previous wave, 5,108 new additions from booster sampling, and 267 unmatched participants or unsolicited opt-ins. The sample retained 4,053 participants from the initial Time 1 (2009) NZAVS of 6,518 participants (a retention rate of 62.2% over three years). The sample retained 5,762 participants from the full Time 3 (2011) sample (a retention rate of 83.7% from the previous year).

Starting from this wave, non-respondents were emailed a follow-up reminder email approximately two months later. Three attempts were then made using each provided phone number (typically home and cell phone) to contact non-respondents to encourage participation. These attempts were made on separate days, approximately one week apart. When possible, a phone message was left for each phone number after the third attempt. Participants were also posted a pamphlet outlining recent findings from the study mid-way through the year. Finally, participants were posted a Season’s Greetings from the NZAVS research team, and informed that they had been automatically entered into a bonus seasonal grocery voucher prize draw for a total pool of $NZ 1,000. Participants were informed that the draw would happen automatically and winners contacted. The Season’s Greetings card also asked participants to contact us (online, email or phone) to let us know if any of their contact details had changed before the prize draw was conducted. These additional materials are available from the NZAVS website [28].

To boost sample size at Time 4 and increase sample diversity for subsequent waves, five independent booster samples using different sample frames were also conducted. Booster sampling was conducted without replacement (i.e., all people included in previous sample frames were identified and removed from the electoral roll before generation of the new sample frames). The first sample frame consisted of a randomly selected sample of 20,000 people from the 2012 New Zealand Electoral Roll (note the electoral roll list all eligible voters, barring those removed on a case-by-case basis due to privacy concerns). A total of 2,429 participants responded to this booster sample (response rate = 12.33% when adjusting for the 98.5% accuracy of the electoral roll in 2012). The second sample frame consisted of a regional booster of 10,000 people randomly selected from people listed in the 2012 Electoral Roll who lived in the Auckland region. A total of 890 participants responded to this booster sample (adjusted response rate = 9.04%). The Auckland region was oversampled because it is the fastest growing and most ethnically diverse region of the country with an increasing number of Asian and Pacific peoples in particular. The questionnaire used for this Auckland sample was longer than the standard NZAVS questionnaire, and contained additional unrelated questions that are not included in the NZAVS dataset (these related to the use of community facilities). (Exit interviews conducted during Time 5 indicated that the longer length of this questionnaire may have contributed to the low response rate in this case.)

The third sample frame consisted of 3,000 people randomly selected from the 2012 Electoral Roll who lived in the Christchurch region. A total of 332 participants responded to this booster sample (adjusted response rate = 11.24%). The Christchurch region was oversampled because it has experienced significant hardship and change due to the Christchurch earthquakes of 2010 and 2011 with many people moving out of the region and problems with mail delivery with some city zones being placed under restricted entry due to safety concerns and considerable infrastructure destroyed.

The fourth sample frame consisted of 9,000 respondents selected from meshblock area units across the country that were moderate-to-high in deprivation [29]. Regions with levels of deprivation were selected using scores on the decile-ranked New Zealand Deprivation Index from 6-10, with 10 being the most deprived. This sample frame used scaled weighting so that people in increasingly derived regions were increasingly more likely to be selected (with random sampling of people within regions that had a given level of deprivation). The scaling factor was as follows: *n_i_* = *n_base_* * *weight_i_*, where *n_base_* = 600, and *weight_i_*, ranged from 1 to 5 and increased by 1 for each one-unit increased in deprivation score. Thus, 600 people were randomly selected from regions with a deprivation score of 6, 1,200 people were randomly selected from regions with a deprivation score of 7, and so on. This sampling strategy was designed to increase the representativeness of the sample across regions with different levels of deprivation, as the NZAVS showed increase in attrition rate in increasingly more deprived regions over the first three years of the study. A total of 767 participants responded to this booster sample (adjusted response rate = 8.65%). The fifth sample frame consisted of 9,000 people randomly selected from those who indicated on the 2012 Electoral Roll that they were of Māori ethnicity (ethnic affiliation as Māori is listed on the roll, but other ethnic affiliations are not). A total of 690 participants responded to this booster sample (adjusted response rate = 7.78%). The questionnaire administered to the Māori booster sample included questions specifically designed for Māori.

***Sampling Procedure -- NZAVS Time 5 (2013).***The Time 5 (2013) NZAVS contained responses from 18,264 participants, including10,502 retained from one or more previous wave, 7,581 new additions from booster sampling, and 181 unmatched participants or unsolicited opt-ins. The sample retained 3,934 participants from the initial Time 1 (2009) NZAVS of 6,518 participants (a retention rate of 60.4% over four years). The sample retained 9,844 participants from the full Time 4 (2011) sample (a retention rate of 80.8% from the previous year). As described in the Time 4 procedure, we offered a prize draw for participation, non-respondents were emailed and phoned multiple times, and all participants were posted a Season’s Greetings card from the NZAVS research team and informed that they had been automatically entered into a bonus seasonal grocery voucher prize draw. We also posted our yearly pamphlet summarizing key research findings published during the current wave of the study.

To boost sample size and increase sample diversity for subsequent waves, two booster samples were also conducted again using the New Zealand Electoral Roll. As with previous booster samples, sampling was conducted without replacement (i.e., all people included in previous sample frames were identified and removed from the 2014 roll). The first sample frame consisted of 70,000 people aged from 18-60 randomly selected from the 2014 New Zealand Electoral Roll. The electoral roll contains participants’ date of birth (within a one-year window), and we limited our frame to people who were 60 or younger, due to our aim of retaining participants for the following 15 years. A total of 7489 participants responded to this booster sample (response rate = 10.9% when adjusting for the 98.6% accuracy of the electoral roll in 2014). The second sample frame consisted of 1,500 people who were listed in the electoral roll as being of Māori ancestry and who were between 18-60 years of age. A total of 92 participants responded to this booster sample (response rate = 6.2% adjusting for electoral roll accuracy).

***Sampling Procedure -- NZAVS Time 6 (2014).***The Time 6 (2014) NZAVS contained responses from 15,822 participants, including 15,740 retained from one or more previous wave, and 82 unmatched participants or unsolicited opt-ins. The sample retained 3,727 participants from the initial Time 1 (2009) NZAVS of 6,518 participants (a retention rate of 57.2% over five years). The sample retained 14,875 participants from the full Time 5 (2012) sample (a retention rate of 81.5% from the previous year). As before, we offered a prize draw for participation, non-respondents were emailed and phoned multiple times, and all participants were posted a Season’s Greetings card from the NZAVS research team and informed that they had been automatically entered into a bonus seasonal grocery voucher prize draw. We also emailed participants an online pamphlet containing a series of video interviews with the researchers summarizing different research findings.

**References**

28. Huang Y, Greaves LM, Sibley CG. Archive of additional (non-questionnaire) materials posted to participants. NZAVS Technical Documents, e07. 2014. Available: [www.psych.auckland.ac.nz/uoa/NZAVS](http://www.psych.auckland.ac.nz/uoa/NZAVS)

29. Salmond C, Crampton P, Atkinson J. NZDep2006 Index of Deprivation. Wellington. Department of Public Health, University of Otago Wellington. 2007. Available: <http://www.otago.ac.nz/wellington/departments/publichealth/research/hirp/otago020194.html>
